# Histidine-Rich Glycoprotein Inhibits High-Mobility Group Box-1-Mediated Pathways in Vascular Endothelial Cells through CLEC-1A

**DOI:** 10.1016/j.isci.2020.101180

**Published:** 2020-05-18

**Authors:** Shangze Gao, Hidenori Wake, Masakiyo Sakaguchi, Dengli Wang, Youhei Takahashi, Kiyoshi Teshigawara, Hui Zhong, Shuji Mori, Keyue Liu, Hideo Takahashi, Masahiro Nishibori

**Affiliations:** 1 Department of Pharmacology, Okayama University Graduate School of Medicine, Dentistry and Pharmaceutical Sciences, 2-5-1 Shikata-cho, Kita-ku, Okayama 700-8558, Japan; 2 Department of Cell Biology, Okayama University Graduate School of Medicine, Dentistry and Pharmaceutical Sciences, Okayama 700-8558, Japan; 3 Department of Pharmacology, School of Pharmacy, Shujitsu University, Okayama 703-8516, Japan; 4 Department of Pharmacology, Faculty of Medicine, Kindai University, Osakasayama 589-8511, Japan

**Keywords:** Molecular Biology, Cell Biology

## Abstract

High-mobility group box-1 (HMGB1) protein has been postulated to play a pathogenic role in severe sepsis. Histidine-rich glycoprotein (HRG), a 75 kDa plasma protein, was demonstrated to improve the survival rate of septic mice through the regulation of neutrophils and endothelium barrier function. As the relationship of HRG and HMGB1 remains poorly understood, we investigated the effects of HRG on HMGB1-mediated pathway in endothelial cells, focusing on the involvement of specific receptors for HRG. HRG potently inhibited the HMGB1 mobilization and effectively suppressed rHMGB1-induced inflammatory responses and expression of all three HMGB1 receptors in endothelial cells. Moreover, we first clarified that these protective effects of HRG on endothelial cells were mediated through C-type lectin domain family 1 member A (CLEC-1A) receptor. Thus, current study elucidates protective effects of HRG on vascular endothelial cells through inhibition of HMGB1-mediated pathways may contribute to the therapeutic effects of HRG on severe sepsis.

## Highlights

• HRG inhibited LPS-induced HMGB1 translocation and release from endothelial cells
• HRG reduced inflammatory responses in endothelial cells caused by released HMGB1
• CLEC-1A was identified as the receptor for the function of HRG on endothelial cells

## Graphical Abstract

**Figure:**
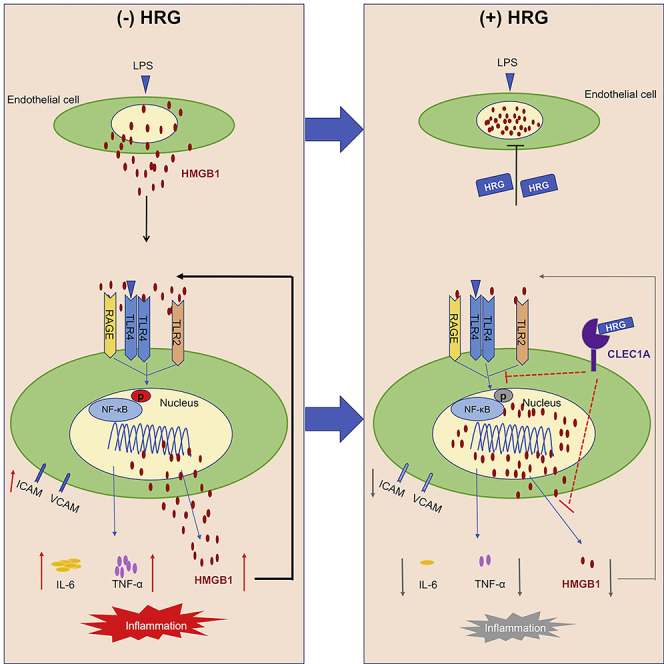

## Introduction

High-mobility group box-1 (HMGB1), a nonhistone chromatin-binding nuclear protein, can be actively secreted into the extravascular space by several immune cell types or passively released by damaged tissues and necrotic cells (DeMarco et al., 2005, Andersson et al., 2000). A high concentration of HMGB1 in the plasma of patients with severe sepsis correlates with a poor prognosis and high mortality, and the pharmacologic inhibition of HMGB1 improved survival in animal models of acute inflammation and severe sepsis (Chen et al., 2004). Systemic inflammation is one of the hallmarks of septic shock, and the disorder of microvascular endothelium appears to produce the amplification of these inflammatory responses (Kirkpatrick et al., 1997). Thus, microvascular injury is one of the characteristics of sepsis-associated tissue damage that may be manifested by single or multiple organ failure syndromes (Lehr et al., 2000; Lentsch and Ward, 2000). HMGB1 was reported to have multiple proinflammatory effects on vascular endothelial cells by binding to three pathogen-associated cell surface pattern recognition receptors, thereby inducing tumor necrosis factor alpha (TNF-α) expression and NF-κB activation in target cells and stimulating the production of an array of proinflammatory cytokines (Abraham et al., 2000, Park et al., 2004, Lotze and Tracey, 2005, Fiuza et al., 2003, Treutiger et al., 2003, Mullins et al., 2004), which suggest an important role for HMGB1 in endothelial cell activation and injury in sepsis and systemic inflammation.

Histidine-rich glycoprotein (HRG) is an abundant plasma protein (60–100 μg/mL) synthesized in liver, which can regulate many biological processes such as angiogenesis, coagulation, and the phagocytosis of apoptotic cells through the interaction with various ligands (Koide et al., 1986, Ronca and Raggi, 2015, Poon et al., 2011). Shannon et al. proved that HRG decreased the mortality of a septic mouse model with *S pyogenes*-induced abscesses by killing and trapping bacteria in the abscess sites (Shannon et al., 2010). Wake et al. reported that HRG prevents septic lethality through the regulation of neutrophils and vascular endothelial cells (Wake et al., 2016). A counteracting role of HRG on damage-associated molecular pattern/pathogen-associated molecular pattern (DAMP/PAMP)-induced responses has long been recognized (Wake et al., 2009, Wake et al., 2016, Gao et al., 2019, Zhong et al., 2018). In recent clinical studies, HRG was also proposed as a new biomarker to predict the outcome of sepsis patients (Kuroda et al., 2018, Nishibori et al., 2018). However, the effect of HRG on HMGB1 release or HMGB1 signaling has never been investigated. It is possible that HRG as a ligand-like molecule may exert its cellular function through the stimulation of unidentified receptors on endothelial cells.

C-type lectin-like receptors (CLECs) comprise a diverse family of transmembrane pattern recognition receptors that are expressed primarily on myeloid cells (Colonna et al., 2000), and CLECs are now considered driving players of sterile inflammation whose dysregulation leads to the development of various pathologies such as autoimmune diseases, allergy, and cancer (Chiffoleau, 2018). CLEC-1A is an orphan type II transmembrane receptor of the C-type lectin superfamily, which is expressed by dendritic cells and endothelial cells in humans (Colonna et al., 2000, Chiffoleau, 2018, Sobanov et al., 2001, Kanazawa, 2007). CLEC-1A triggering may directly modulate the activation of the dendritic cells and endothelial cells and/or send a regulatory signal to T cells (Sattler et al., 2012, Lopez Robles et al., 2017). Therefore, CLEC-1A may be a useful target to modulate immune responses toward protective immunity.

In present study, we firstly proved that the inhibitory effects of HRG on lipopolysaccharide (LPS)-induced HMGB1 translocation and HMGB1-induced signal pathway were mediated through the stimulation of specific receptors of CLEC-1A on vascular endothelial cells. Our results strongly suggest that the inhibition of HMGB1 action by HRG may contribute to HRG's mortality-reducing protective activity against severe sepsis, and our findings also further explained the mechanism underlying the regulation of neutrophils and endothelium barrier function by HRG under septic conditions *in vivo* and *in vitro* (Wake et al., 2016, Gao et al., 2019). This demonstration of an intimate relationship between HRG and HMGB1 provides direct supporting evidence for the supplementary treatment of sepsis with HRG.

## Results

### HRG Inhibited LPS-induced HMGB1 Translocation and Release in Endothelial Cells

**Figure 1:**
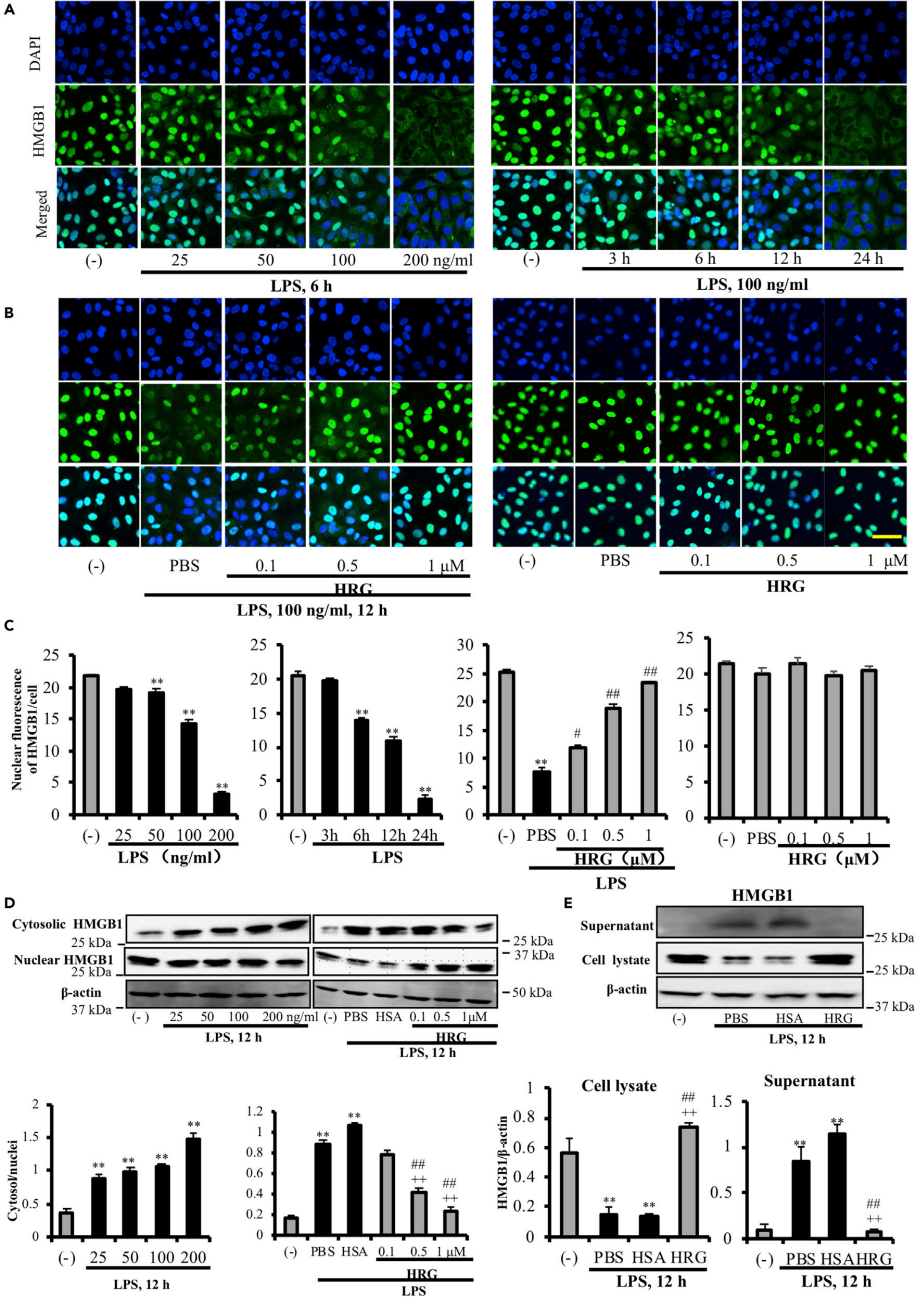
HRG Inhibited LPS-induced HMGB1 Translocation and Release in EA.hy926 Cells (A) EA.hy926 cells were stimulated with the indicated concentrations of LPS for indicated time, and the translocation of HMGB1 was observed by immunostaining as described in the Methods section. HMGB1 staining (*green*) and nucleus staining (*blue*) fluorescence are shown. (B) EA.hy926 cells were incubated with different concentrations of HRG or PBS for 1 h before being stimulated with 100 ng/mL LPS for 12 h. See also Figure S1–S3. (C) The quantification results of nuclear HMGB1 using ImageJ software. The results shown are the means ± SEM of five determinations. (D) EA.hy926 cells were stimulated with the indicated concentrations of LPS for 12 h (*left panel*), and the HMGB1 in the cytosol and nucleus was determined by western blotting. The LPS (100 ng/mL)-induced HMGB1 translocation was determined under different concentrations of HRG (*right panel*). The cytosolic and nuclear extracts were prepared with NE-PER extraction reagents, and the content of HMGB1 was measured by western blotting. The results were quantified by ImageJ software and are expressed as the ratio of cytosol/nuclei HMGB1. (E) Endothelial cells were cultured for 12 h with LPS (100 ng/mL) in the presence or absence of HRG. The cell lysates and the supernatants were collected and analyzed for HMGB1 by western blotting. See also Figure S4. All results are the means ± SEM of five different experiments. Scale bars, 5 μm. One-way ANOVA followed by the post hoc Fisher test. ^∗∗^p < 0.01 versus control, ^##^p < 0.01 and ^++^p < 0.01 versus PBS and HSA.

Several studies showed that HMGB1 can be released from human endothelial cells in response to both endotoxin and TNF-α (Fiuza et al., 2003, Treutiger et al., 2003, Mullins et al., 2004). EA.hy926 is a transformed cell line, established by fusing primary human umbilical vein endothelial cells with a thioguanine-resistant clone of A549, carcinoma of human alveolar epithelial cell. Our results showed that LPS actively induced HMGB1 translocation from EA.hy926 cells in a concentration- and time-dependent manner (Figure 1A), whereas HRG inhibited this process in a concentration-dependent manner (Figure 1B, left panel). However, HSA, a major plasma protein, did not show any inhibitory effects at the same concentration (1 μmol/L). In addition, HRG did not show any influence on HMGB1 translocation in the absence of LPS (Figure 1B, right panel). These results were quantified with ImageJ software through the setting of a cutoff level of HMGB1 fluorescence in the cell nuclei (Figure 1C). HRG also inhibited the TNF-α-induced HMGB1 translocation in EA.hy926 cells (Figure S1). The inhibition effect of HRG on LPS-induced HMGB1 translocation was also true in primary human lung microvascular endothelial cells (HMVECs, Figure S2).

To confirm these results, we also measured the HMGB1 content in nucleus, cytoplasma, and culture medium of endothelial cells with western blotting. The results showed that HRG not only inhibited HMGB1 translocation from nucleus to cytoplasma (Figure 1D) but also effectively suppressed HMGB1 release into extracellular space (Figure 1E). The cell viability assay also proved that the LPS stimulation triggered an active release of HMGB1 from EA.hy926 cells and both LPS (100 ng/mL) and rHMGB1 (1 μg/mL) were not toxic to the endothelial cells (Figure S3). In addition, HRG effectively inhibited the LPS-induced decrease in HMGB1 mRNA expression in both EA.hy926 cells and HMVECs (Figure S4).

### HRG Suppressed the rHMGB1-Mediated Adhesive Molecule Expression and Neutrophil Adhesion

**Figure 2:**
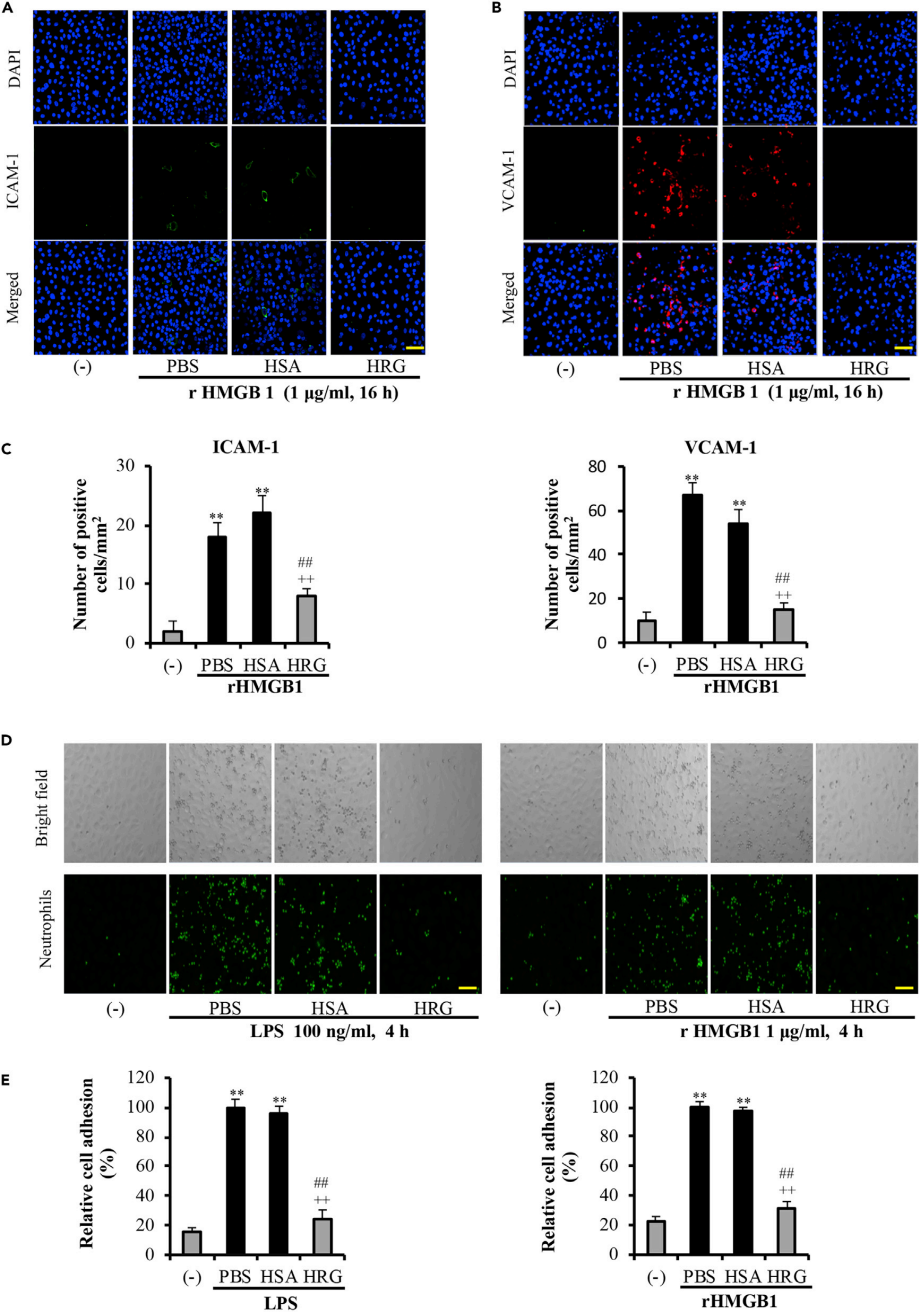
HRG Suppressed the rHMGB1-Mediated Adhesive Molecule Expression and Neutrophils Adhesion on EA.hy926 Cells (A and B) Confluent EA.hy926 cells were incubated with rHMGB1 (1 μg/mL for 16 h) after being treated with HRG or HSA for 1 h. The cell surface expression of (A) VCAM-1 (*green*) and (B) ICAM-1 (*red*) on the cells was observed by immunostaining. See also Figure S5. (C) Positive cells were counted in six fields of each group. (D) A confluent endothelial monolayer was incubated with LPS (100 ng/mL) or rHMGB1 (1 μg/mL) in the presence or absence of HRG, and the amount of neutrophils that adhered to the endothelial monolayer was measured. (E) The data are the percentage of neutrophil numbers compared with those in PBS. The results shown are the means ± SEM of three experiments. Scale bars, 20 μm. One-way ANOVA followed by the post hoc Fisher test. ^∗∗^p < 0.01 versus control, ^##^p < 0.01 and ^++^p < 0.01 versus PBS and HSA.

HMGB1 was reported to induce inflammatory responses by increasing the cell surface expressions of the cell adhesion molecules ICAM-1, VCAM-1, and E-selectin on the surface of endothelial cells, thereby promoting the adhesion and migration of leukocytes across the endothelium and to sites of inflamed tissues (Fiuza et al., 2003, Treutiger et al., 2003, Mullins et al., 2004). As presented in Figures 2A–2C, HRG effectively inhibited rHMGB1-induced expressions of ICAM-1 and VCAM-1. These effects of HRG were also observed in primary human lung microvascular endothelial cells (Figure S5).

As is well known, elevated expressions of adhesion molecules correlate well with the enhanced binding of neutrophils to endothelial cells and their subsequent migration. We therefore performed cell adhesion experiments using purified neutrophils and endothelial cell co-culture system. The results showed that HRG inhibited the adhesion of human neutrophils to both LPS- and rHMGB1-activated endothelial cells (Figures 2D and 2E).

### HRG Prevented the rHMGB1-induced Inflammatory Responses

**Figure 3:**
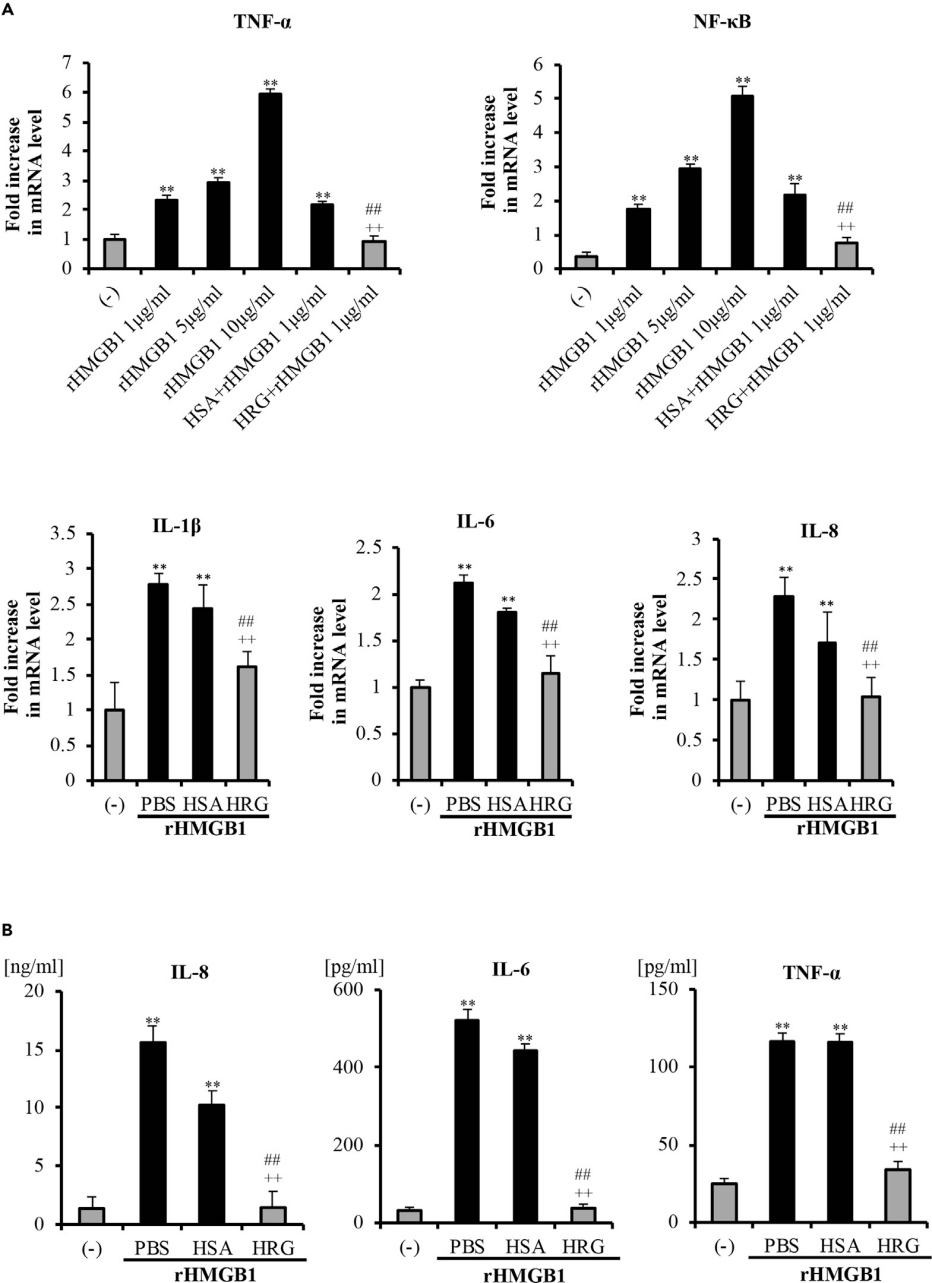
HRG Inhibited the rHMGB1-Stimulated Expression and Secretion of Proinflammatory Mediators in Endothelial Cells EA.hy926 cells were cultured with rHMGB1 (1 μg/mL) for 12 h in the presence or absence of HRG (1 μM). (A) The mRNA expressions of TNF-α, NF-κB, IL-1β, IL-6, and IL-8 in the cells were measured by quantitative RT-PCR. The results were normalized to the expression of β-actin and are expressed as the means ± SEM (n = 5 per group). (B) Cell-free supernatants were harvested after 12-h stimulation with rHMGB1 to measure the concentrations of different proinflammatory cytokines by a CBA assay. The levels of IL-6, IL-8, and TNF-α in the culture media from each group are shown as the mean ± SEM (n = 5 per group). One-way ANOVA followed by the post hoc Fisher test. ^∗∗^p < 0.01 versus control, ^##^p < 0.01 and ^++^p < 0.01 vs. PBS and HSA.

**Figure 4:**
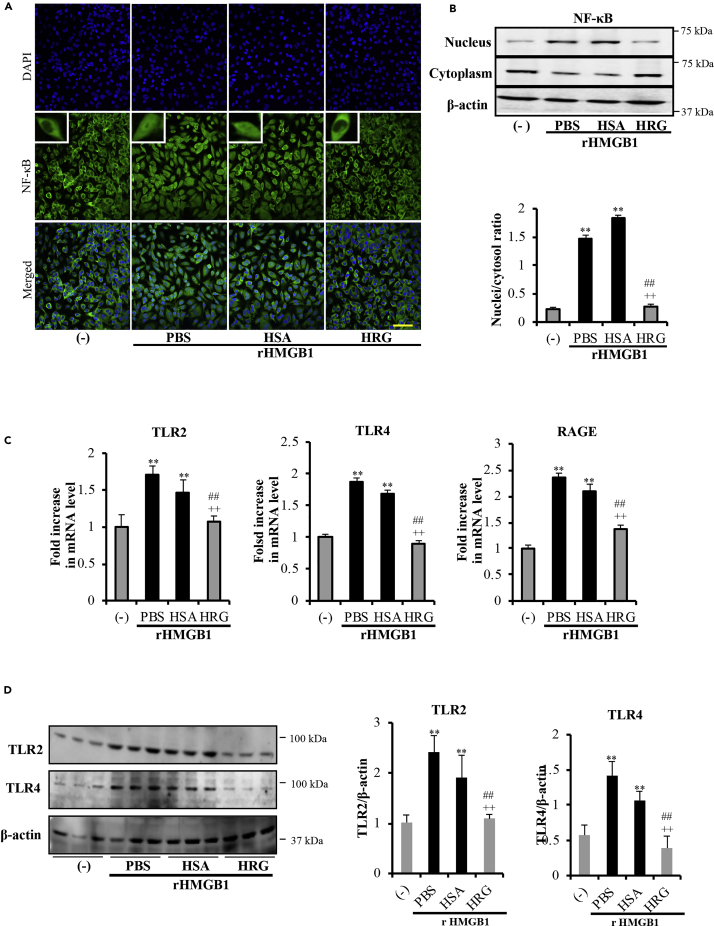
HRG Suppressed the rHMGB1-induced NF-κB Activation and Receptors Expressions in Endothelial Cells (A) Immunostaining results of NF-κB in endothelial cells stimulated with 1 μg/mL rHMGB1 for 6 h after treatment with HRG or HSA. Cells were stained with anti-NF-κB/p65 mAb for 2 h and then stained with Alexa Fluor 488 (*green*)-labeled goat-anti-rabbit IgG. Cells were also stained with DAPI (*blue*) to visualize the nuclei. The results are representative of ≥5 experiments. Scale bar, 10 μm. (B) Cytosolic and nuclear extracts were prepared with NE-PER extraction reagents, and the levels of NF-κB/p65 were determined by western blotting. The results were quantified by ImageJ software. (C) Confluent endothelial cells were incubated with rHMGB1 (1 μg/mL for 16 h) with or without pretreatment with HRG (1 μM) for 1 h. The expressions of TLR2, TLR4, and RAGE in the cells were measured by RT-PCR. The results were normalized to the expression of β-actin and are expressed as the means ± SEM (n = 5 per group). (D) The protein levels of these receptors were also determined by western blotting. The results were quantified with ImageJ software. All results are means ± SEM of five different experiments. One-way ANOVA followed by the post hoc Fisher test. ^∗∗^p < 0.05 versus control, ^##^p < 0.05 and ^++^p < 0.05 versus PBS and HSA.

Cytokine overproduction is one of the major events in severe inflammatory responses. Released HMGB1 is known to interact with specific cell receptors to amplify inflammatory responses by inducing the expression of proinflammatory cytokines (Fiuza et al., 2003, Treutiger et al., 2003, Mullins et al., 2004). According to RT-PCR results in Figure 4A (upper panel), the 12-h incubation of EA.hy926 cells with different concentrations of rHMGB1 resulted in concentration-dependent expressions of TNF-α and NF-κB. HRG (1 μM) strongly inhibited rHMGB1 (1 μg/mL)-induced responses. In addition, HRG significantly inhibited the rHMGB1-induced increase in the expressions of IL-6, IL-8, and IL-1β (Figure 3A) and cytokine secretion in the culture medium (Figure 3B).

HMGB1 was reported to upregulate inflammatory pathways by activating NF-κB and promoting the expression of TNF-α by endothelial cells and monocytes (Andersson et al., 2000). As presented in Figure 4A, NF-κB/p65 was localized mainly in the cytoplasm in the non-stimulated control group, whereas in the rHMGB1-treated cells, NF-κB/p65 was translocated into the nuclei. Pretreatment with HRG clearly suppressed the rHMGB1-induced NF-κB/p65 translocation to nuclei in endothelial cells in immunostaining and western blotting (Figures 4A and 4B). The effects of HRG on the NF-κB activation were consistent with HRG's effects on the rHMGB1-induced cytokine production.

### HRG Downregulates the Expression of HMGB1 Receptors

The pro-infiammatory activity of rHMGB1 was reported to be mediated via its interaction and subsequent signaling through TLR2, TLR4, and RAGE (Park et al., 2004). As shown in Figure 4C, rHMGB1 induced the mRNA expression of all three receptors in endothelial cells by 1.7- to 2.4-fold, and HRG significantly inhibited the stimulatory effects of rHMGB1 on the expressions of TLR2, TLR4, and RAGE. Similar effects on their protein levels were also shown by western blotting (Figure 4D). These results demonstrated that one of the action mechanisms of HRG depends mainly on the suppression of the receptors' overexpression on endothelial cells induced by rHMGB1.

### HRG Inhibited HMGB1 Release and HMGB1-Mediated Inflammatory Responses *In Vivo*

**Figure 5:**
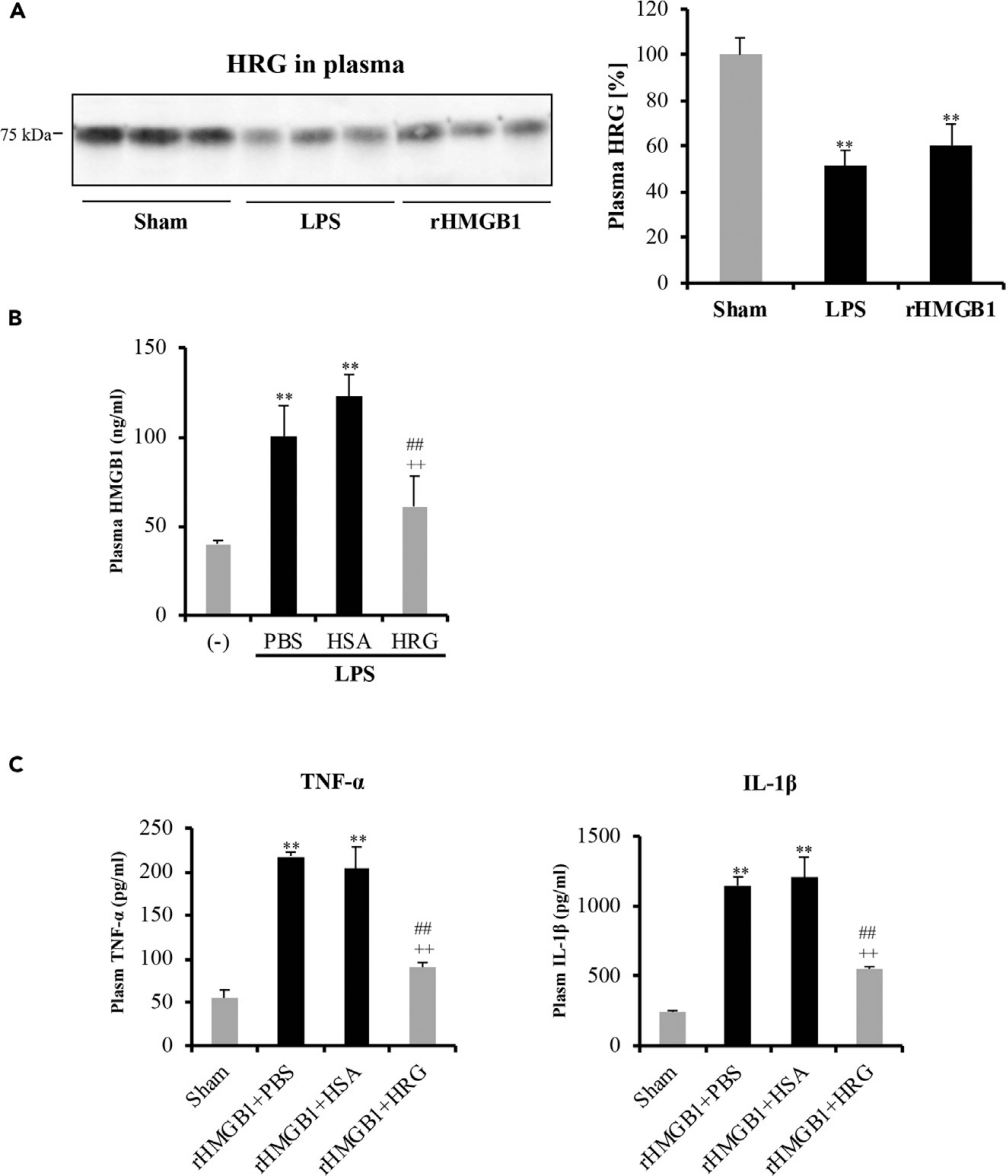
HRG Inhibited HMGB1 Release and HMGB1-Mediated Inflammatory Responses In Vivo C57BL/6N mice was intravenous injected of LPS (10 mg/kg) or recombinant HMGB1 (100 μg) and then HRG or HSA in vehicle (PBS) was administered through the tail vein immediately. Each mouse was given 20 mg/kg HRG or HSA in a volume of 200 μL (IV). After 12 h the whole blood from mouse heart was taken and centrifuged for 10 min at 3,000 rpm to obtain the plasma. (A) Plasma levels of HRG in mice were determined 12 h after LPS (10 mg/kg) or rHMGB1 (100 ug/mouse) injection by western blotting. The relative expression levels were calculated as percentage of sham control. (B) HMGB1 levels in plasma were measured in LPS-injected mice (12 h) by ELISA. (C) Proinflammatory cytokine TNF-α and IL-1β were measured 12 h after rHMGB injection in mice with cytokine beads assay. N = 6, one-way ANOVA followed by the post hoc Fisher test. ^∗∗^p < 0.05 versus Sham, ^##^p < 0.05 and ^++^p < 0.05 versus PBS and HSA.

To further confirm the relationship of HRG and HMGB1, we also examined the role of HRG on the regulation of HMGB1 release and rHMGB1-mediated signals *in vivo*. Previous study showed that plasma HRG dramatically decrease in septic or LPS-induced endotoxemia mice (Wake et al., 2016). Our results in Figure 5A proved that not only LPS- but also rHMGB1-injected mice showed obvious reduction of plasma HRG in mice. HMGB1 is a late mediator of endotoxin lethality in mice (Wang et al., 1999). Supplementary treatment with HRG in LPS-injected mice showed inhibition effects on the production of plasma HMGB1 (Figure 5B). Moreover, HRG significantly suppresses the elevated proinflammatory cytokine TNF-α and IL-1β in rHMGB1-injected mice (Figure 5C). These results proved that HRG also can regulate the HMGB1 release and HMGB1-mediated inflammatory responses *in vivo*.

### CLEC-1A Was Identified as the Receptor for HRG

**Figure 6:**
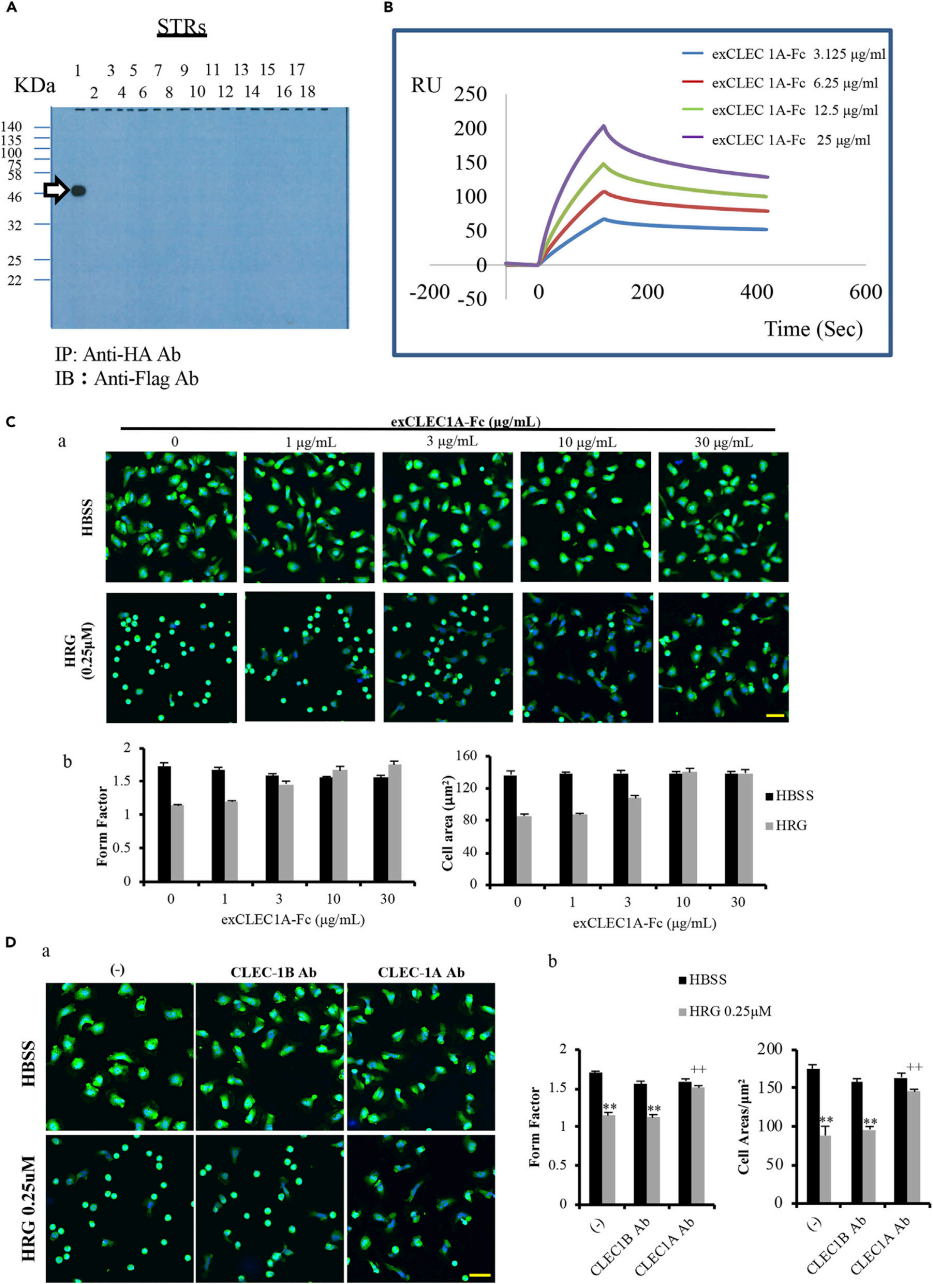
The Identification of CLEC-1A Receptor for HRG (A) HEK293T cells were transiently transfected with the plasmid vector of maHRG combined with each vector encoding a series of collected receptors, using FuGENE-HD. The collected receptors contained CLEC-1A, -1B, -2A, -2B, -2C, -2D, -4A, -4C, -4D, -4E, -4F, -4G, -4M, -5A, -6A, -7B, -12A, and -12B. After 24 h of the transfection, cell pellets were prepared and lysed by M-PER mammalian protein extraction reagent. The lysates were then incubated with agarose beads conjugated with monoclonal anti-HA tag antibody to pull-down the expressed maHRG. The resulting immunoprecipitates were then subjected to western blotting using monoclonal anti-Flag tag antibody to detect maHRG-bound receptor candidate(s). (B) The binding affinity of CLEC-1A-Fc to HRG *in vitro*. Purified HRG (5 μg/mL) from human plasma was immobilized on a CM5 BIAcore chip, and different concentrations of the extracellular domain of CLEC-1A-Fc (exCLEC-1A-Fc) fusion protein (3.125, 6.25, 12.5, or 25 μg/mL) were flowed at time zero for 120 s. Surface plasmon resonance (BIAcore) showed a rapid increase in response units (RU), indicating the binding of exCLEC-1A-Fc fusion protein to the immobilized HRG. The K_D_ for exCLEC-1A-Fc binding to HRG was determined as 7×10^−9^ M. See also Figure S6. (C and D) Purified human neutrophils were labeled with calcein-AM (*green*) and Hoechst33342 (*blue*) for 20 min. The neutrophils were incubated with HBSS or HRG (0.25 μM) together with different concentrations of exCLEC-1A-Fc fusion protein (1–30 μg/mL) or 10 μg/mL CLEC-1A Ab or CLEC1B Ab for 1 h, and the neutrophil shapes were observed by fluorescence microscopy. (a) Typical picture of fluorescence staining from each group; scale bars, 20 μm. (b) The shapes and sizes of neutrophils were analyzed by an IN Cell Analyzer 2000. The Form factor (max. dia./min. dia.) and cell area (mm^2^) were determined. One unit represents an ideal spherical shape of neutrophils in the Form factor. The results are the means ± SEM of five experiments. ^∗∗^p < 0.01 versus control and ^++^p < 0.01 versus CLEC1B Ab.

In light of the multi-gene families of candidate receptors, we selected 18 genes (*CLEC-1A*, -*1B*, -*2A*, -*2B*, -*2C*, -*2D*, -*4A*, -*4C*, -*4D*, -*4E*, -*4F*, -*4G*, -*4M*, -*5A*, -*6A*, -*7B*, -*12A*, and -*12B*) from the CLEC family, two genes (*TREM-1* and *-4*) from the TREM (triggering receptor expressed on myeloid cells) family, and five genes (*SIGLEC-3*, *-5*, *-9*, *-14*, *-15*) from the SIGLEC (sialic acid-binding Ig type lectin [SIGLEC]) family in accord with our interest in HRG binding. The individual genes were examined for HRG interaction. After the dual transfection of maHRG with each receptor plasmid to HEK293T cells, we examined the maHRG co-immunoprecipitates from the transfected cell lysates by western blotting to evaluate the potential binding of maHRG with the foreign receptors. The results indicated that one event of interaction clearly occurred in the screening trial using a CLEC family member, i.e., CLEC-1A (Figure 6A). We observed no appreciable interaction with the TREM or SIGLEC family members (data not shown). Collectively, these results prompted us to focus on CLEC-1A as a novel receptor for HRG.

The BIAcore sensograms of both exCLEC-1A-Fc and human recombinant exCLEC-1A protein to immobilized HRG showed a rapid increase in response units (RUs), indicating the binding of both exCLEC-1A-Fc fusion protein and human recombinant exCLEC-1A protein to the immobilized HRG on the chip, followed by a decrease in the RU resulting from the dissociation of binding molecules upon washing. The binding of exCLEC-1A-Fc fusion protein and human recombinant exCLEC-1A protein to HRG was concentration dependent (Figures 6B and S6A). The equilibrium dissociation constant (K_D_) was determined as 7×10^−9^ M and 4×10^−7^, respectively. These results thus suggested a high-affinity binding of HRG to CLEC-1A. Meanwhile, when the exCLEC-1A-Fc fusion protein was immobilized on the CM5 sensor chip, the BIAcore sensogram of HRG to immobilized exCLEC-1A-Fc also showed a concentration-dependent increase in response units (Figure S6B). The equilibrium dissociation constant (K_D_) was determined as 1×10^−8^ M. These results demonstrate a high-affinity binding of HRG to CLEC-1A in both directions.

### Blocking of CLEC-1A Neutralizes the Protective Activity of HRG on Neutrophils and Endothelial Cells

It was reported that HRG (1 μmol/L) maintained the spherical shape of neutrophils, sustaining the rheological stability and preventing the unnecessary activation of vascular endothelial cells (Wake et al., 2016). We observed the morphological changes of the neutrophils under a fluorescent microscope at 60 min after incubation without a fixation procedure. Compared with the HBSS group, these neutrophils treated with HRG had a spherical shape, a loss of the irregularity of shapes, and shortened diameters. However, the spherical shape-inducing effects of HRG were significantly neutralized by the treatment with exCLEC-1A-Fc fusion protein (Figure 6C-a) or CLEC-1A Ab (Figure 6D-a), accompanied with increase of Form factor and cell area (Figures 6C-b and 6D-b).

**Figure 7:**
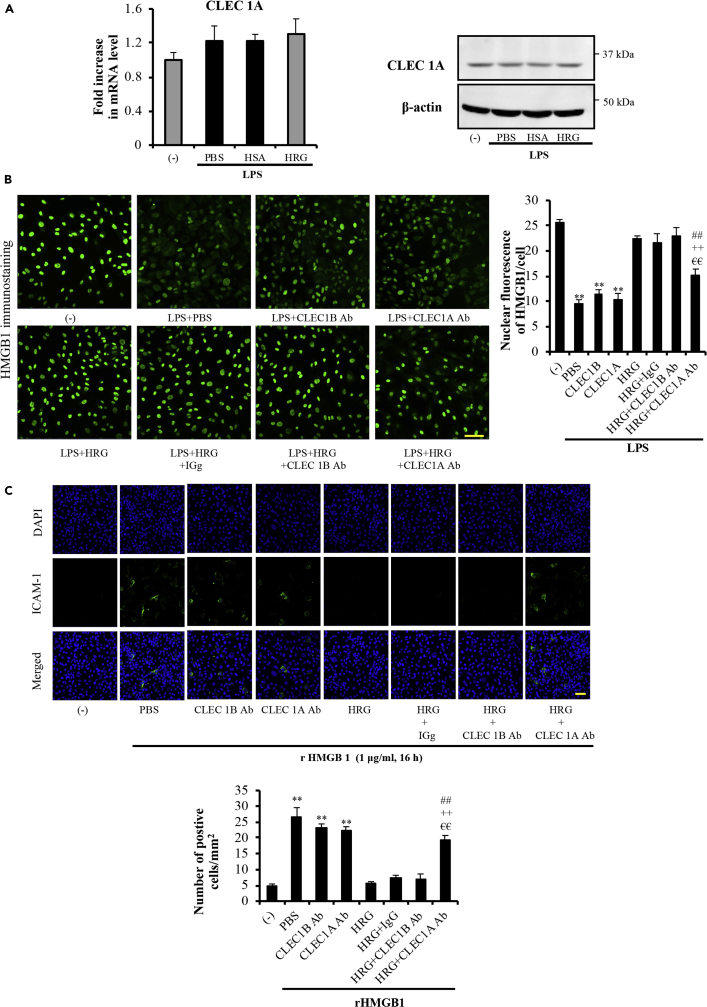
The Inhibitory Effects of Anti-CLEC-1A Antibody on the Protective Activity of HRG on the EA.hy926 Cells (A) CLEC-1A expression on EA.hy926 cells was confirmed with western blotting and RT-PCR at the protein and mRNA levels. (B, C) EA.hy926 cells were cultured for 12 h until confluence and then pre-incubated with 10 μg/mL CLEC-1A Ab or CLEC1B Ab or goat IgG control antibody in DMEM medium for 6 h. The cells were then stimulated with LPS for 12 h in the presence or absence of HRG (1 μM). The immunostaining of HMGB1 (B) and ICAM-1 (C) were performed as described in the Methods section. A representative picture of fluorescence staining from each group is shown; scale bars, 20 μm. The quantitative results in the graphs are means ± SEM (n = 5 per group). One-way ANOVA followed by the post hoc Fisher test. ^∗∗^p < 0.01 versus control, ^##^p < 0.01, ^++^p < 0.01 and ^€€^p < 0.01 versus HRG, control IgG, and CLEC1B Ab group, respectively.

The western blotting and RT-PCR results established that CLEC-1A was expressed in EA.hy926 cells. The expression level of CLEC-1A was not changed after stimulation with LPS (Figure 7A). The experiment results in Figure 7 confirmed that the inhibitory effects of HRG on the LPS-induced HMGB1 translocation and rHMGB1-stimulated ICAM-1 expression in EA.hy926 cells were clearly neutralized by the pre-incubation with CLEC-1A Ab (10 μg/mL) for 6 h (Figures 7B and 7C). In addition, CLEC-1A or CLEC-1B Ab alone did not show any effects on the LPS-induced activation of endothelial cells (Figures 7B and 7C) or regulation of morphology changes of neutrophils (Figure 6D). These results provide evidence that CLEC-1A may be one of the receptors of HRG′ s action on neutrophils and endothelial cells.

## Discussion

HMGB1 plays a novel inflammatory cytokine-like role that contributes to the lethality of sepsis, and neutralizing of HMGB1 release protected animals from the lethality of endotoxemia (Sama et al., 2004, Wang et al., 1999, Wang et al., 2001). The results presented herein together with those reported by others (Fiuza et al., 2003, Treutiger et al., 2003, Mullins et al., 2004) suggest that vascular endothelial cells may be rich sources of HMGB1. Released HMGB1 activates endothelial cells by upregulating its surface receptors. It was also reported that recombinant HMGB1 had potent proinflammatory activity on vascular endothelial cells (Bae and Rezaie, 2011, Degryse et al., 2001). Therefore, the modulation of the release of HMGB1 from endothelial cells and the regulation of released HMGB1-mediated signal pathways in endothelial cells may provide a novel means of treating acute inflammatory conditions, especially in sepsis.

Clinical studies revealed that the plasma HRG levels of septic patients were significantly lower than those of healthy subjects (Kuroda et al., 2018, Nishibori et al., 2018). Wake et al. demonstrated that supplementary treatment with HRG can effectively improve the survival rate of septic mice and relieve their inflammatory syndrome (Wake et al., 2016). Our previous study established that HRG has a potent protective activity on vascular endothelium barrier function under septic condition (Gao et al., 2019). Nevertheless, the effects of HRG on HMGB1 release and HMGB1-mediated proinflammatory responses in vascular endothelial cells had not been studied prior to the present investigation.

Our present findings showed that HRG effectively inhibited LPS-induced HMGB1 translocation from nucleus to the cytoplasm and subsequently to the extracellular space (Figure 1). Although HMGB1 can be passively released after necrosis, our results demonstrated that the LPS-induced translocation and release of HMGB1 in EA.hy926 cells is independent of cell death. We did not detect any significant increase in cell apoptosis or necrosis during the stimulation with LPS (Figure S3). In addition, HRG effectively inhibited the LPS-induced decrease in HMGB1 mRNA expression in both EA.hy926 cells and HMVECs (Figure S4). HRG alone did not induce any changes in the HMGB1 mRNA expression in the absence of LPS stimulation (Figure S4). Vascular endothelial cells regulate the inflammatory process through the expression of adhesion molecules, cytokines, chemokines, and growth factors (Tedgui and Mallat, 2001, Davies et al., 1993, Tedgui and Mallat, 2006). Endothelial cell adhesion molecules such as VCAM-1 and ICAM-1 are upregulated in vascular endothelial cells in response to inflammatory stimuli, which in turn mediates leukocyte recruitment and adherence onto the endothelium (Blankenberg et al., 2003, Figueras-Aloy et al., 2007). The released HMGB1 can further activate endothelial cells, leading to the upregulation of cell adhesion molecules, i.e. ICAM-1, VCAM-1, and E-selectin (Fiuza et al., 2003, Treutiger et al., 2003). Herein we proved HRG also downregulated the rHMGB1-mediated expression of the cell surface adhesion molecules ICAM-1 and VCAM-1 (Figures 2A–2C), thereby inhibiting the adhesion of the neutrophils to the activated endothelial cells (Figures 2D and 2E). Previous studies' experiments established that HRG effectively regulated neutrophils' activation and immunothrombosis formation in septic mice, and our present results confirmed that HRG can control the interaction between neutrophils and endothelial cells from both sides.

The inhibition of the production of pro-inflammatory cytokines is a key factor in the prevention and therapy of sepsis. Extracellular HMGB1 can activate endothelial cells through the activation of NF-κB, leading to the release of proinflammatory cytokines such as TNF-α and IL-1β (Cohen, 2002). Our results clearly demonstrated that treatment with HRG can strongly inhibit the rHMGB1-enhanced expression/secretion of IL-8, IL-6, and TNF-α (Figure 3) and NF-κB activation (Figures 4A and 4B). These findings proved that HRG regulate not only the HMGB1 release but also the subsequent inflammatory process induced by extracellular HMGB1 on endothelial cells. HMGB1 upregulated proinflammatory responses by interacting with three pathogen-related pattern recognition receptors: TLR2, TLR4, and RAGE. HRG effectively suppressed the cell surface expression of all three HMGB1 receptors (Figures 4C and 4D) in endothelial cells. These results provide a likely explanation of how HRG effectively reduces the inflammatory response in rHMGB1-activated endothelial cells.

HMGB1 was recognized as a late mediator of sepsis, and the neutralization of HMGB1 or the inhibition of its release protected animals from the lethality of endotoxemia (Sama et al., 2004, Wang et al., 1999, Wang et al., 2001). Our results show that both LPS and rHMGB1 injection can induce the reduction of HRG in mice plasma (Figure 5A), suggesting the LPS- or rHMGB1-induced liver injury that influence the HRG production (Tsung et al., 2005, Gong et al., 2010). The supplementary treatment with HRG can effectively inhibit the HMGB1 release in LPS-injected mice and reduce inflammatory cytokine production in rHMGB1-injected mice (Figures 5B and 5C). These results provide a new evidence that HRG improves the survival rate, and relieve of inflammatory symptom of septic mice may be associated with the regulation of HMGB1 signaling.

A counteracting role of HRG on DAMP/PAMP-induced responses has long been recognized (Wake et al., 2016, Kuroda et al., 2018, Nishibori et al., 2018). It is possible that HRG as a ligand-like molecule may exert its cellular function through the stimulation of unidentified receptors. We thus first examined DAMP-related receptors, i.e., macrophage-inducible C-type lectin (MINCLE) (also known as C-type lectin-like receptor 4E [CLEC4E]) (Yamasaki et al., 2008), and triggering receptor expressed on myeloid cells-1 (TREM-1) (El Mezayen et al., 2007), because ligands for these receptors have not been comprehensively identified, unlike those of the well-known DAMP receptors, toll-like receptor 4 (TLR4), and RAGE. We also investigated SIGLEC family receptors that are key modifiers of neutrophil functions (McMillan et al., 2013, Favier, 2016). Based on our screening trial using CLEC family members, CLEC-1A was observed to be the only one among a set of candidate receptor molecules that interacts with the membrane-anchored HRG that we designed to be expressed on the cell surface (Figure 6A). The results of our surface plasmon resonance experiments demonstrated that exCLEC-1A-Fc binds to HRG with high affinity (K_D_ value of 7×10^−9^ M) (Figure 6B); meanwhile, HRG binds to immobilized exCLEC-1A-Fc with K_D_ value of 1×10^−8^ M (Figure S6B). These results provide strong evidence that CLEC-1A is one of the receptors for HRG. To further confirm these results, we examined the protective effect of HRG on neutrophils and endothelial cells after blocking the CLEC-1A. The results showed that the spherical shape-inducing effects of HRG were significantly neutralized by the treatment with exCLEC-1A-Fc fusion protein (Figure 6C-a) or CLEC-1A Ab (Figure 6D-a). Moreover, pretreatment with CLEC-1A antibody effectively prevented the inhibiting effects of HRG on LPS-induced HMGB1 translocation (Figure 7B) and HMGB1-mediated ICAM-1 expression (Figure 7C) in EA.hy926 cells. The neutralizing effect of exCLEC-1A-Fc fusion protein and the blocking effects of CLEC-1A antibody on the action of HRG on EA.hy926 cells and neutrophils further indicate that CLEC-1A may be a novel receptor for HRG.

It is worthy of mentioning that CLEC-1A might not be the sole receptor for HRG. Our previous study proved that HRG augmented natural killer cell function by modulating PD-1 expression via CLEC-1B (Nishimura et al., 2019). Stanniocalcin-2 (STC2) was also reported as an interacting partner of HRG on the surface of inflammatory cells *in vitro*. The colocalization of HRG and STC2 in gliomas may play a role for suppressing glioma growth by modulating tumor inflammation through monocyte infiltration and differentiation (Roche et al., 2018). However, whether the interaction of HRG with STC2 may influence the HRG-CLEC-1A-mediated signaling need to be clarified in the future. HRG is a multidomain protein consisting of 2 N-terminal regions, a central histidine-rich region (HRR) and a C-terminal domain. HRG interacts with many ligands through various binding domains, regulating a number of biological processes. Some ligands binding to the HRR domain of HRG is dependent on Zn^2+^ or acidic pH (Sundberg and Martin, 1974;; Morgan, 1981). It should be addressed in the future study, whether HRG-CLEC1A binding is modulated by Zn^2+^.

The present study is the first report that HRG efficiently inhibited LPS-induced HMGB1 translocation and release and the HMGB1-mediated signal pathway in vascular endothelial cells through CLEC-1A receptor. These results added strong evidence to our previous observation that supplementary HRG treatment in septic mice can relieve the septic syndrome and prevent endothelium barrier dysfunction. The role of HRG in barrier protection may depend mainly on its effect of inhibiting HMGB1 release from endothelial cells. Our results thus not only clarify the receptor of the effects of HRG on vascular endothelial cells but also provide evidence for HRG as a treatment for sepsis.

### Limitation of the Study

We proved the role of HRG on the regulation of HMGB1 signaling in endothelial cells through CLEC-1A receptor using *in vitro* experiments. In addition, our previous study found that the knockdown of liver HRG by siRNA could exacerbate septic inflammation and lethality compared with that in the control mice, which indicated that the depletion of HRG are more vulnerable to sepsis (Wake et al., 2016). However, the involvement of HRG and CLEC-1A in septic condition are not clarified and characterized *in vivo* in current research. Hrg−/− and Clec-1A−/− mice would be used in the future research. Moreover, although we proved the binding between CLEC 1A and HRG *in vitro*, the binding domain on HRG and the relationship with Zn^2+^ and PH will require additional studies.

### Resource Availability

#### Lead Contact

Further information and requests for resources and reagents should be directed to and will be fulfilled by the Lead Contact, Masahiro Nishibori (mbori@md.okayama-u.ac.jp).

#### Materials Availability

All unique reagents generated in this study are available from the Lead Contact with a completed Materials Transfer Agreement.

#### Data and Code Availability

This study did not generate/analyze (datasets/code).

## Methods

All methods can be found in the accompanying Transparent Methods supplemental file.

## Supplemental Information

Document S1. Transparent Methods, Figures S1–S6, and Table S1
