# Supplementary material for: Histidine-Rich Glycoprotein Inhibits High-Mobility Group Box-1-Mediated Pathways in Vascular Endothelial Cells through CLEC-1A
Source: iScience. 2020 May 18;23(6):101180. doi: 10.1016/j.isci.2020.101180 (PMC7267745; doi:10.1016/j.isci.2020.101180)
Supplement: Document S1. Transparent Methods, Figures S1–S6, and Table S1 [file mmc1.pdf]

iScience, Volume *23*

Supplemental Information

Histidine-Rich Glycoprotein Inhibits High-Mobility Group Box-1-Mediated Pathways in Vascular Endothelial Cells through CLEC-1A

Shangze Gao, Hidenori Wake, Masakiyo Sakaguchi, Dengli Wang, Youhei Takahashi, Kiyoshi Teshigawara, Hui Zhong, Shuji Mori, Keyue Liu, Hideo Takahashi, and Masahiro Nishibori

Supplemental Figures

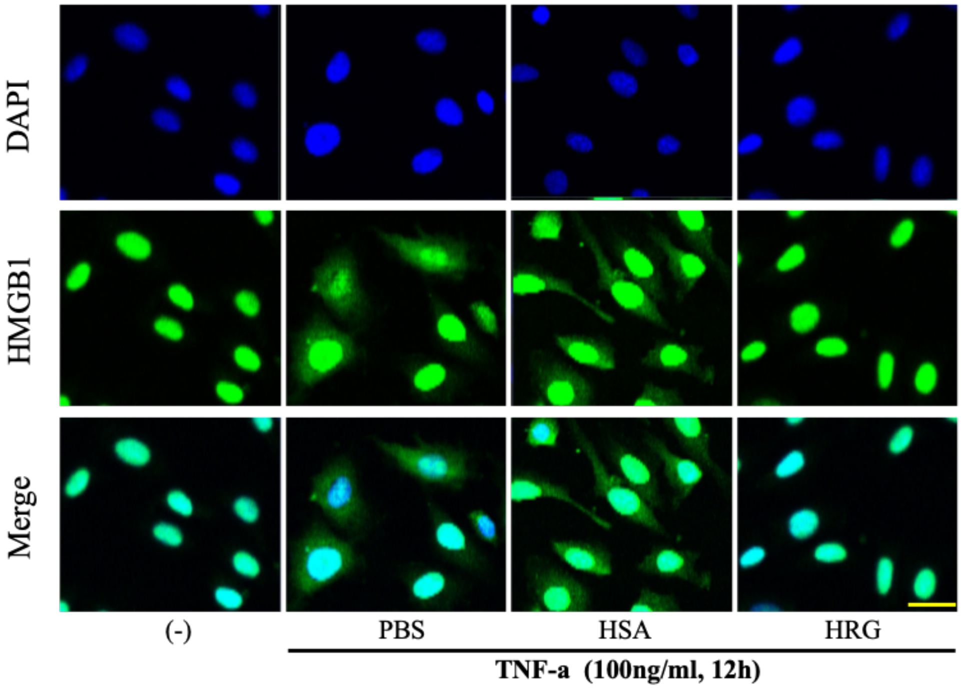

Figure S1. The effects of HRG on the TNF-α-induced HMGB1 translocation in EA.hy 926 cells, related to Figure 1. EA.hy 926 cells were incubated with HRG or phosphate-buffered saline (PBS) for 1 h before being stimulate with 100 ng/ml TNF-α for 12 h, and the translocation of HMGB1 was observed by immunostaining as described in the Methods section of the main text. HMGB1 staining (*green*) and nucleus staining (*blue*) are shown. Images are representative of three independent experiments. Scale bar = 20 μm.

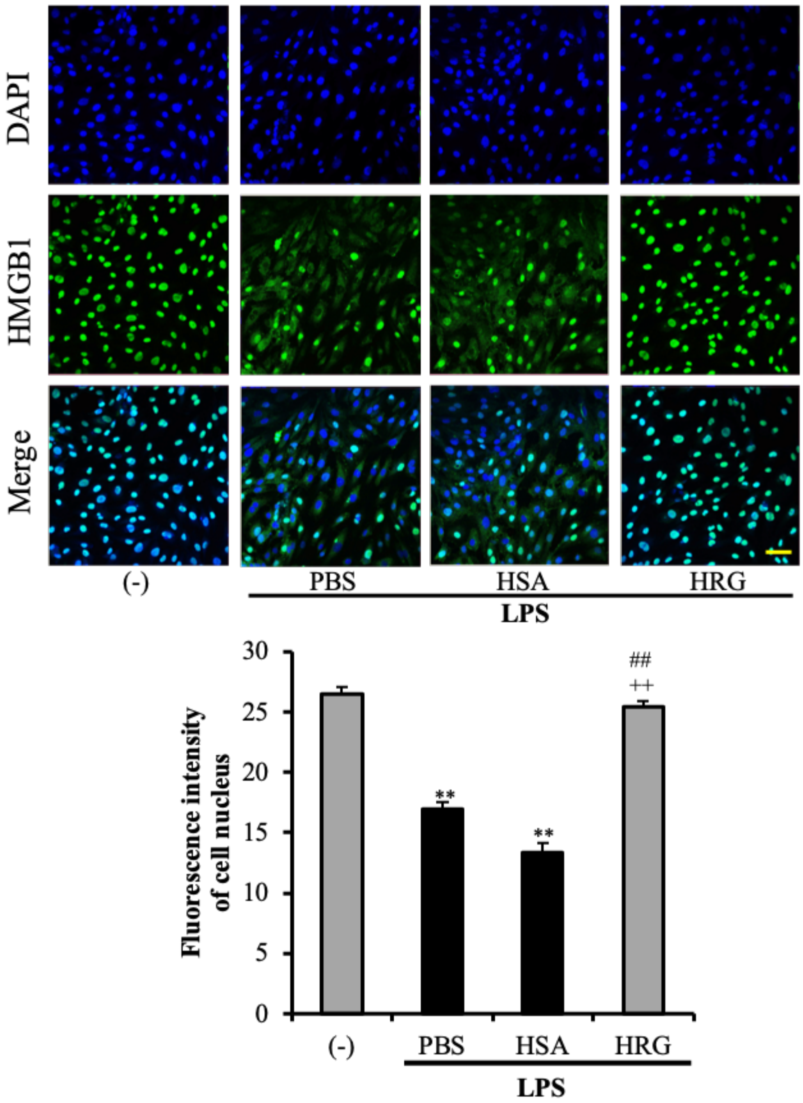

Figure S2. The effects of HRG on the LPS-induced HMGB1 translocation in HMVECs, related to Figure 1. Human primary lung microvascular endothelial cells (HMVECs) were incubated with HRG or PBS for 1 h before being stimulated with 100 ng/ml LPS for 12 h, and the translocation of HMGB1 was observed by immunostaining as described in the Methods section. HMGB1 staining (*green*) and nucleus staining (*blue*) are shown. Images are representative of three independent experiments. Scale bar = 20 μm. The nuclear HMGB1 was quantified using ImageJ software. The graph results are means ± SEM (n=5 per group). One-way ANOVA followed by the post hoc Fisher test. $^{**}p<0.01$ vs. control, $^{\#\#}p<0.01$ and $^{++}p<0.01$ vs. PBS.

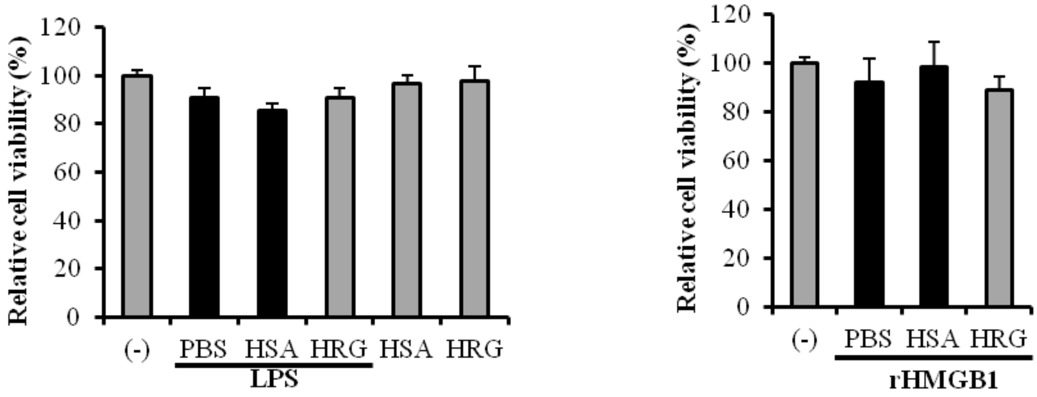

Figure S3. Cell viability of EA.hy 926 cells after stimulated with LPS or rHMGB1, related to Figure 1. EA.hy 926 cells were pre-incubated with HRG/HSA for 1 h before stimulation with LPS (100 ng/ml) or rHMGB1 (1 μg/ml) for 8 h. The cells were then incubated with MTT at 37℃ for 4 h by adding 10 μl of 5 ng/ml MTT solution into each well. After the removal of the cell supernatant, 200 μl of DMSO was added into each well to dissolve the crystals. The OD value was recorded using microplate reader at 570 nm wavelength. All results are the means ± SEM of three different experiments.

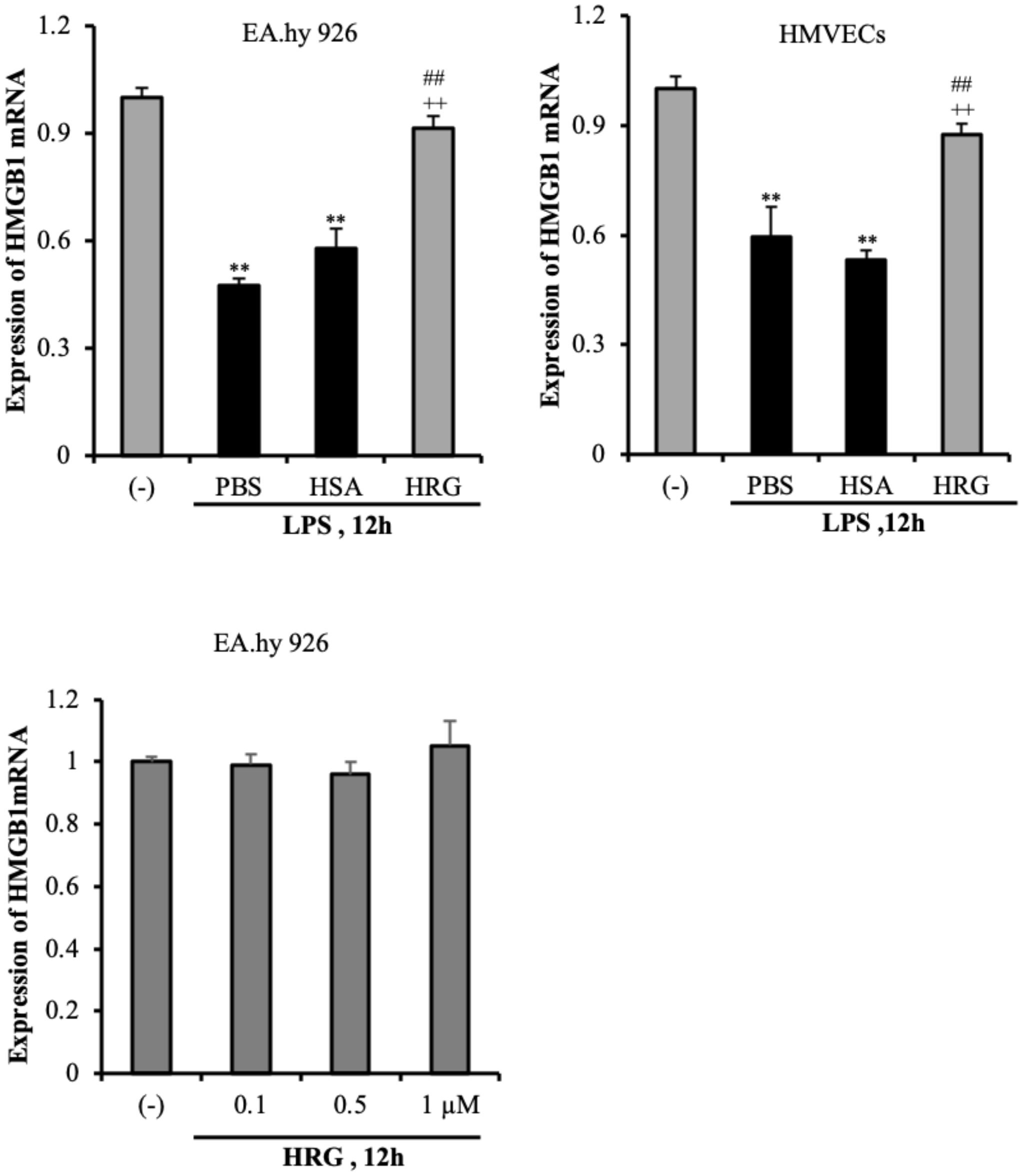

Figure S4. HRG inhibited the reduction of HMGB1 mRNA expression induced by LPS stimulation in EA.hy 926 cells and HMVECs, related to Figure 1. Both lines of vascular endothelial cells were cultured with LPS in the presence or absence of HRG. The expression of HMGB1 at the mRNA level on the cells was measured by quantitative RT-PCR. The results were normalized to the expression of β-actin and are expressed as the means ± SEM (n=5 per group). One-way ANOVA followed by the post hoc Fisher test. **$p<0.01$ vs. control, $^{\#\#}p<0.01$ and $^{++}p<0.01$ vs. PBS and HSA.

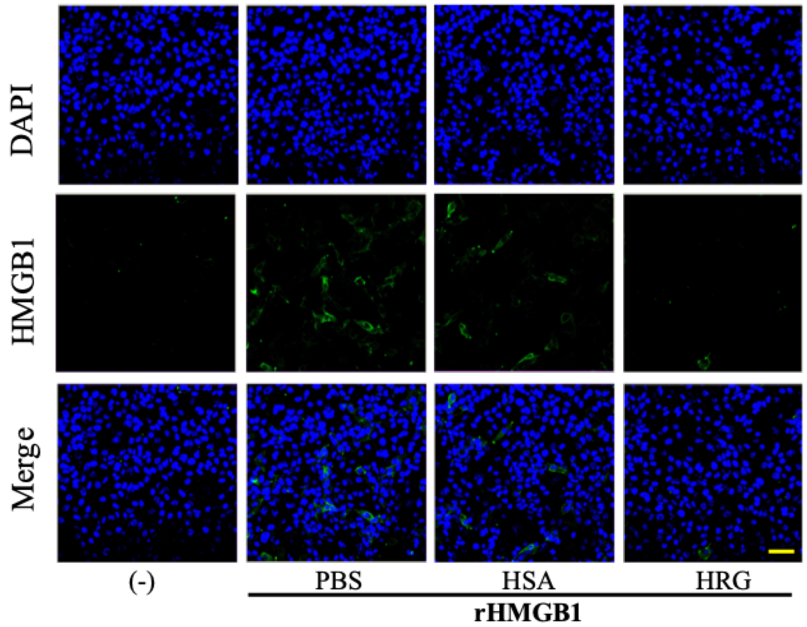

Figure S5. Effect of HRG on the rHMGB1-induced expression of cell adhesion molecules in HMVECs, related to Figure 2. Confluent endothelial cells were incubated with rHMGB1 (1 μg/ml for 16 h) after being treated with the indicated concentrations of HRG or HSA for 1h. The cell surface expression of ICAM-1 (*green*) on HMVEC was observed by immunostaining. Scale bars = 20 μm.

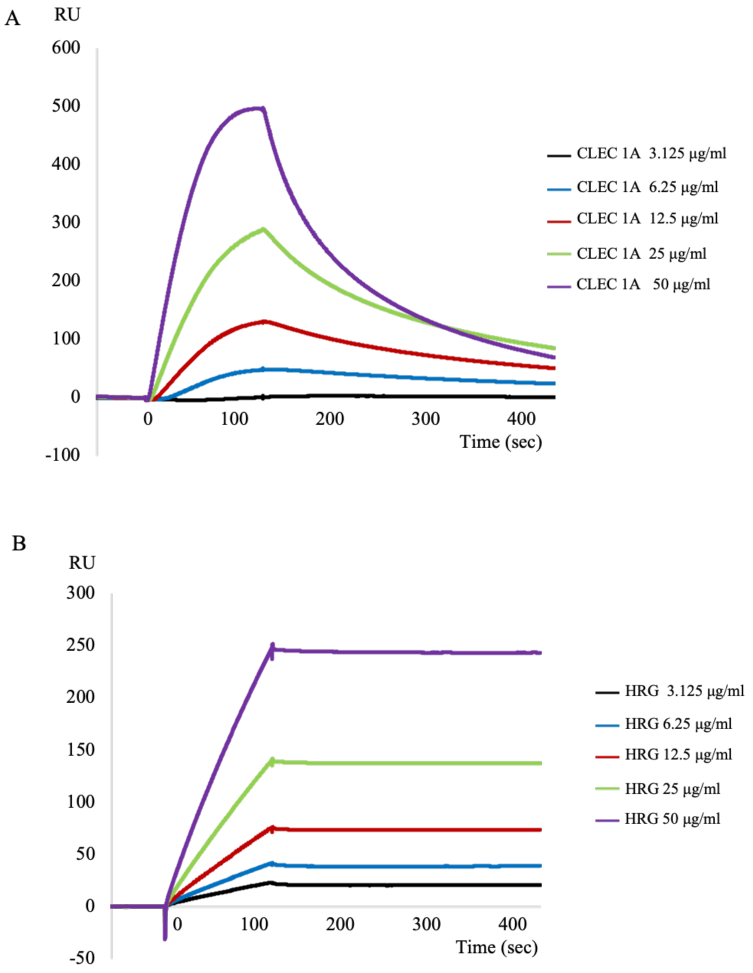

Figure S6. HRG and CLEC 1A interaction in vitro unsing Biacore T200, related to Figure 6. **A:** The binding affinity of human recombinant exCLEC-1A protein to HRG in vitro. Purified HRG (5 µg/ml) from human plasma was immobilized on a CM5 BIAcore chip, and different concentrations of the human recombinant exCLEC-1A protein (3.125, 6.25, 12.5, 25 or 50 µg/ml) were flowed at time zero for 120 sec. Surface plasmon resonance (BIAcore) showed a rapid increase in response units (RU), indicating the binding of human recombinant CLEC-1A protein to the immobilized HRG. The K_D value was determined as 4×10^{-7} M. **B:** The binding affinity of HRG to exCLEC-1A-Fc in vitro. Extracellular domain of CLEC-1A-Fc (exCLEC-1A-Fc) fusion protein (10 µg/ml) was immobilized on a CM5 sensor chip, and different concentrations of the purified HRG from human plasma (3.125, 6.25, 12.5, 25 or 50 µg/ml) were flowed at time zero for 120 sec. Surface plasmon resonance (BIAcore) showed a rapid increase in response units (RU), indicating the binding of HRG to the immobilized exCLEC-1A-Fc fusion protein. The K_D for HRG binding to exCLEC-1A-Fc was determined as 1×10^{-8} M.

Table S1. RT-PCR primer sequences, related to Figure3, Figure 4, Figure 7 and transparent methods.

mRNA	Sense primer	Anti-sense primer
β-actin:	5'-AGCGGGAAATCGTGCGTG-3'	5'-CAGGGTACATGGTGGTGCC-3'
IL-6:	5'-GAACTCCTTCTCCACAAGCGCCTT-3'	5'-CAAAAGACCAGTGATGATTTTCACCAGG-3'
IL-8:	5'-ATGACTTCCAAGCTGGCCGTGGCT-3'	5'- TCTCAGCCCTCTTCAAAAACTTCTC-3'
IL-1β:	5'-CAGCCATGCCAGAAGTACCT-3'	5'-GACATCACCAAGCTTTTTTGC-3'
TNF-α:	5'-GAGTGACAAGCCTGTAGC-3'	5'-CCCTTCTCCAGCTGGAAG-3'
NF-κBp65:	5'-GCCATGGACGAACTGTTCCCC-3'	5'-GGGAA CAGTTCGTCCATGGC-3'
TLR-2:	5'-GCCAAAGTCTTGATTGATTGG-3'	5'-TTGAAGTTCTCCAGCTCCTG-3'
TLR4:	5'-ACTCCCTCCAGGTTCTTGATTAC-3'	5'-CGGGAATAAAGTCTCTGTAGTGA-3'
RAGE:	5'-GCCCTCCAGTACTACTCTCG-3'	5'-TGTGTGGCCACCCATTCCAG-3'
CLEC-1A:	5' –AAACAAGAAGACCTGGAATTTGC-3'	5'- TCTTGGGCTGGTGACATCTATTA-3'
HMGB1:	5'-AGATATGGCAAAAGCGGACAAG-3'	5'-TCAGAGCAGAAGAGAAGAAGG-3'

Transparent Methods

Cell cultures

EA.hy 926 cell line (ATCC Cat# CRL-2922, RRID:CVCL_3901), a hybridoma of human umbilical vein endothelial cells (HUVECs) and the adenocarcinomic human alveolar basal epithelial cells A549, were cultured using Dulbecco's modified Eagle medium (DMEM, #D6546, Sigma, St. Louis, MO) supplemented with 10% fetal bovine serum (Gibco, Grand Island, NY), 5% L-glutamine (#G7513, Sigma), and 10% penicillin/streptomycin (Gibco) in 5% CO_2 at 37°C. After reaching confluence, the EA.hy 926 cells were detached from culture flasks using 0.25% Trypsin-EDTA (Gibco), washed, and resuspended in DMEM. These cells were passaged every 3–4 days, and all experiments were performed with the cells kept in culture between three and six passages as described (Wake et al., 2016; Gao et al., 2019).

Primary human lung microvascular endothelial cells (HMVECs) were obtained from Lonza (#CC-2527; Walkersville, MD). HMVECs were cultured in EBMTM-2 Basal Medium (#CC-3156, Lonza) with the recommended supplements in the EGMTM-2MV SingleQuots Kit (#CC-4147, Lonza) in 5% CO_2 at 37°C. After reaching confluence, the endothelial cells were detached from culture flasks with Accutase® (10 ml per 75 cm^2) at 37°C for 10 min, washed, and resuspended in culture medium. These cells were passaged every 3–4 days, and all experiments were performed with the cells kept in culture between three and ten passages.

Purification of HRG from human plasma

HRG was purified from human plasma by our lab as described (Mori et al., 2003). Human plasma was supplied by the Japanese Red Cross Society from the healthy volunteer's donation. The study protocol complied with the principles outlined in the Declaration of Helsinki and all subjects signed an informed consent. Briefly, human plasma was incubated with nickel-nitrilotriacetic acid (Ni-NTA) agarose (Qiagen, Hilden, Germany) for 2 h at 4°C with gentle shaking. The gel was packed into a column and washed successively with 10 mmol/L Tris-buffered saline (TBS) (pH 8.0) containing 10 mmol/L imidazole and then 10 mmol/L Tris-buffer (TB) (pH 8.0) containing 1 mol/L NaCl. Human HRG was eluted by 0.5 mol/L imidazole in 10 mmol/L TBS (pH 8.0). The protein eluate from Ni-NTA was further purified by a Mono Q column (GE Healthcare, Little Chalfont, UK) with NaCl gradient. Purified human HRG was identified by sodium dodecyl sulfate-polyacrylamide gel electrophoresis (SDS-PAGE) and Western blotting with a human HRG-specific antibody.

Immunostaining assay

EA.hy 926 cells or HMVEC cells were pretreated with various concentrations of HRG or human serum albumin (HSA) for 1 h before stimulated with LPS (Escherichia coli 0111:B4, Sigma) (25–200 ng/ml) or human recombinant HMGB1 (rHMGB1, Abnova, Taiwan) (1 µg/ml). The cells were then fixed with 4% paraformaldehyde (Wako Pure Chemical Industry, Osaka, Japan) and blocked with 10% bovine serum albumin (BSA), after which the cells were stained by anti-HMGB1 Ab (rabbit, Sigma, RRID:AB_444360), anti-NF-κB p65 Ab (rabbit, Abcam, RRID:AB_443394), anti-ICAM-1 Ab (Ms, Sigma, RRID:AB_445260), or anti-VCAM-1 Ab (#ab134047, Sigma, RRID:AB_2721053) for 1 h at 37°C followed by Alexa Fluor 488/568-labeled anti-rabbit/mouse

IgG. Cell nuclei were stained with DAPI for 5 min, and then observed using a confocal microscope (LSM 780, Carl Zeiss).

Western blotting

The whole cell lysate was collected with RIPA lysis buffer (50 mM Tris-HCl, 150 mM NaCl, 1% NP-40, 0.5% sodium deoxylcholate, 0.1% SDS, 1 mM EDTA, 1 mM DTT, 20 mM β-glycerophosphate, and protease/phosphatase inhibitors added immediately before use) and the cytoplasmic and nuclear extracts were collected with NE-PER Nuclear and Cytoplasmic Extraction Reagents (#78833, ThermoScientific, Rockford, IL). The mice plasma sample was collected with sample buffer. All the samples were then electrophoresed on polyacrylamide gels and transferred onto a polyvinylidene difluoride (PVDF) membrane (Bio-Rad, Hercules, CA). The membrane was blocked with 10% skim milk for 1 h and incubated with rabbit anti-HMGB1 Ab (Rb, #ab18256, Sigma), anti-TLR2 Ab (Rb, #ab191458, Abcam), anti-TLR4 Ab (Rb, Abcam, RRID:AB_10561435), anti-CLEC 1 Ab (goat, #AF1704, RRID:AB_2083452), anti-HRG Ab (Rb, #GTX64492, Gen Tex) and anti-β-actin Ab (#sc-47778, Santa Cruz, RRID:AB_2714189) followed by goat anti-rabbit IgG-HRP (MBL, Nagoya, Japan) for 2 h at room temperature. The signals were visualized by the enhanced chemiluminescence HRP substrate method (Thermo Fisher Scientific, Waltham, MA). An Image Quant LAS4000 system was used for detection, and images were analyzed with ImageJ software ver. 1.51.

Cell viability

EA.hy 926 cells were plated in 96-well plates at $5×10^5$ overnight, and then pre-incubated with HRG/HSA for 1 h before stimulation with LPS (100 ng/ml) or rHMGB1 (1 μg/ml) for 8 h. The cells were then incubated with MTT at 37℃ for 4 h by adding 10 μl of 5 ng/ml MTT solution into each well. After the removal of the cell supernatant, 200 μl of DMSO was added into each well to dissolve the crystals. The absorbance of each well was measured using a microplate reader (model 680, Bio-Rad) at 570 nm wavelength, and the optical density (OD) value was recorded.

Isolation of neutrophils

Human neutrophils were isolated from peripheral blood obtained from healthy volunteers in accordance with ethics approval and guidelines of Okayama University and the Declaration of Helsinki. The blood was drawn from the antecubital vein. Human polymorphonuclear neutrophils (PMNs) were isolated by density gradient centrifugation over Polymorphprep™ (Axis-Shield, Oslo, Norway). Briefly, blood was layered over an equal volume of Polymorphprep and centrifuged at 500 g for 45 min at 22°C. The lower band containing neutrophils was subsequently collected and washed with PBS by centrifugation at 400 g for 10 min. The cells were counted with a hemocytometer (EKDS, Tokyo) by trypan blue dye exclusion. After the centrifugation, the pellet was resuspended in Hank's balanced salt solution (HBSS). Purified human neutrophils were labeled with calcein-AM (green) and Hoechst 33342 (blue) for 20 min at 37°C and then washed once with PBS. Finally, the cells were resuspended in HBSS to a final concentration of $2×10^6$ cells/ml.

Neutrophil adhesion assay

EA.hy 926 cell suspensions ($5×10^5$ cells/ml) were cultured in 96-well plates for 16 h until confluent for the cell adhesion assay. The monolayer was then washed with PBS and pretreated with HRG or HSA (1 μmol/L) for 1 h before being stimulated with LPS (100 ng/ml) or rHMGB1 (1 μg/ml) at 37°C in a 5% CO_2 atmosphere for 4 h. After the incubation, the cells were washed with PBS. The pre-labeled neutrophils ($1×10^6$ cells/ml) were then added to the stimulated endothelial monolayer and co-cultured for 1 h. The neutrophils were allowed to become adherent, and the nonadherent neutrophils were washed off. The fluorescence of the adherent cells was then measured. The percentage of adherent neutrophils was calculated as: the percentage of adherence = (adherent signal/total signal) ×100, as described (Bae et al., 2011).

RNA isolation and RT-PCR

EA.hy 926 cells or HMVEC cells were harvested and mRNA was extracted using an RNeasy mini kit (Qiagen). Complementary DNA was synthesized with a Takara RNA PCR kit ver. 3.0 (Takara Bio, Nagahama, Japan) and RT-PCR was performed with a Light Cycler (Roche, Basel, Switzerland) according to the manufacturer's instructions. The primers shown in Supplemental Table S1 were used to amplify specific cDNA fragments. The β-actin expression was used to normalize the cDNA levels.

Mice

Adult male C57BL/6N mice (22±3 g, 8 week, RRID: MGI_5658420) were purchased from SLC (Hamamatsu, Japan) and then housed in the Okayama University institutional animal units (12 h light cycle). All animal experiments were approved by the university's committee and performed according to the guidelines of Okayama University on animal experiments. The C57BL/6N mice was intravenous injected of LPS (10 mg/kg) or recombinant HMGB1 (100 μg) and then HRG or HSA in vehicle (PBS) was administered through the tail vein immediately. Each mouse was given 20 mg/kg HRG or HSA in a volume of 200 μl (i.v.). After 12 h the whole blood from mouse heart were taken and used for the following experiment (Edward et al., 2000, Gao et al. 2019).

Ezyme-linked Immunosorbent Assay (ELISA)

To determine HMGB1 levels in plasma, blood samples were collected through the mouse heart under deep anesthesia, then centrifuged for 10 min at 3000 rpm. HMGB1 was detected by using an ELISA kit (Shino-Test Co, Sagamihara, Japan), according to the manufacturer's instructions.

Cytometric bead array (CBA)

We measured the secreted cytokines in the supernatant of cultured medium or plasma form rHMGB1-injected mice by performing CBA using a Human Soluble Protein Master Buffer Kit and cytokine Flex Set (#558264, BD Biosciences, San Jose, CA) following the manufacturer's instructions. Generally, multiple capture beads for interleukin (IL)-6, IL-8, and TNF-α were mixed together. The mixed capture beads were co-incubated with 50 μl of supernatant and detection reagent for 2 h. The beads were then washed carefully and resuspended. Samples were analyzed using a FACSCanto II system (BD Biosciences). The data were analyzed with FCAP Array

software.

Plasmid constructs

We hypothesized that if we could force the secreted extracellular HRG to specifically concentrate on the cell surface at a much higher level, such abundant HRG on the plasma membrane will readily recognize and bind with its specific receptor(s). Based on this hypothesis, we designed HRG to express on the cell surface, resulting in the construction of modified HRG which we named membrane-anchored HRG (maHRG). This modified HRG has a single-pass transmembrane (TM) domain sourced from basal cell adhesion molecule (BCAM) at the C-terminus of HRG protein.

In order to efficiently express the transgenes that include maHRG and a set of candidate receptor molecules in a transient manner, we inserted cDNAs of our interest into the pIDT-SMART (C-TSC) vector (Sakaguchi et al., 2014). The prepared cDNAs were as follows: human cDNAs encoding maHRG and collected CLEC family receptors (CLEC-1A, -1B, -2A, -2B, -2C, -2D, -4A, -4C, -4D, -4E, -4F, -4G, -4M, -5A, -6A, -7B, -12A and -12B). The modified maHRG was further designed to be expressed in a C-terminal HA form. The CLEC receptors were all designed for their expression as C-terminal 3xFlag-6His-tagged forms.

Immunoprecipitation

We used HEK293T cells for the co-immunoprecipitation experiments. The cells were transiently transfected with the plasmid vector of maHRG combined with each vector containing a series of collected receptors, using FuGENE-HD (Promega, Madison, WI). After 24 h of the transfection, cell pellets were prepared and lysed by *M-PER* mammalian protein extraction reagent (Thermo Fisher Scientific). The lysates were then incubated with agarose beads conjugated with monoclonal anti-HA tag antibody (Sigma-Aldrich, St. Louis, MO) to pull-down the expressed maHRG. The resulting immunoprecipitates were subsequently subjected to western blotting using monoclonal anti-Flag tag antibody (Sigma-Aldrich) to detect maHRG-bound receptor candidate(s).

Surface plasmon resonance analysis (BIAcore)

The extracellular domain of CLEC-1A (exCLEC-1A)-Fc fusion protein was prepared from its corresponding conditioned media from cultures of a FreeStyle™ CHO-S cell (Chinese hamster ovary cell subline; Thermo Fisher Scientific)-derived stable clone. The stably expressed clone for exCLEC-1A-Fc was established by a convenient electroporation gene delivery method using our original pSAKA-1B vector as described (Kinoshita et al., 2019). After the collection of the serum-free conditioned medium from a large-scale culture of the established CHO cell clone, recombinant exCLEC-1A-Fc protein was purified by protein-G affinity chromatography according to the manufacturer's instructions.

We analyzed the binding affinity of exCLEC-1A-Fc fusion protein or human recombriant exCLEC-1A (1704-CL-050, R&D system) to HRG with the use of a BIAcore T200 instrument (GE Healthcare, GE Healthcare Life Sciences, Piscataway, NJ). First, HRG (5 μg/ml) diluted by sodium acetate buffer (pH 5.0) was immobilized on the sensor chip (Biacore sensor chip CM5). An adjacent vacant flow-cell was activated with equal amounts of 0.2 M N-ethyl-N-[3-diethylamino-

propyl]-carbodiimide and 0.05 M N-hydroxysuccinimide under the same conditions as a negative control. HBS-EP + buffer was used for the sample dilution and analysis. exCLEC-1A-Fc fusion protein at a series of concentrations (3.125, 6.25, 12.5, and 25 μg/ml) was passed over the surface sensor chip at a flow rate of 30 μl/min for 2 min, and then dissociation was allowed by the application of HBS-EP buffer. The sensor chips were regenerated by washing with 10 mM glycine-HCl (pH 2.5) for 60 sec at a flow rate of 10 μl/min.

We also analyzed the binding affinity of HRG to the immobilized exCLEC-1A-fc fusion protein. exCLEC-1A-fc fusion protein (10 μg/ml) diluted by sodium acetate buffer (pH 5.0) was immobilized on the sensor chip. Purified HRG (3.125, 6.25, 12.5, 25 and 50 μg/ml) was passed over the surface sensor chip at a flow rate of 30 μl/min for 2 min, and then dissociation was allowed by the application of HBS-EP buffer. The sensor chips were regenerated by washing with 10 mM glycine-HCl (pH 2.5) for 60 sec at a flow rate of 10 μl/min. The results were calculated after the subtraction of the control values using BIAcore evaluation T200 software.

Effects of HRG on neutrophils and EA.hy 926 cells after CLEC-1A blocking

Prelabeled neutrophils were incubated with HBSS or HRG (0.25 μM) together with different concentrations of exCLEC-1A-Fc fusion protein or 10 μg/ml anti-human CLEC-1A or CLEC-1B goat polyclonal antibody (R&D Systems, Minneapolis, MN) for 1 h at 37°C. The shapes of the neutrophils were then observed under a fluorescence microscope. The cell shapes and cell sizes were analyzed by using an IN Cell Analyzer 2000 (GE Healthcare/Life Sciences, Tokyo) and IN Cell Analyzer Workstation software (GE Healthcare/Life Sciences) as described (Wake et al., 2016). The form factor (max. dia./min. dia.) and the cell area were determined in each group.

EA.hy 926 cells were pre-incubated with 10 μg/ml CLEC-1A Ab, CLEC1B Ab and goat polyclonal control IgG antibodies in DMEM medium for 6 h. The cells were then stimulated with LPS for 12 h in the presence or absence of HRG (1 μM). Immunostaining of HMGB1 and intercellular adhesion molecule (ICAM)-1 were performed as described above.

Statistical analysis

The data were analyzed with GraphPad Prism software ver. 6.01 (San Diego, CA). All values are presented as the mean ± SEM and were analyzed by an analysis of variance (ANOVA) followed by Bonferroni's test or post hoc Fisher test when the F statistic was significant. Probability (p) values <0.05 were considered significant. At least three independent experiments were performed for all of the assays.

Supplemental References:

Kinoshita R, Sato H, Yamauchi A, Takahashi Y, Inoue Y, Sumardika IW, Youyi C, Nahoko T, Kota A, Kazuhiko S., et al. (2019) exSSSRs (extracellular S100 soil sensor receptors) – Fc fusion proteins work as prominent decoys to S100A8/A9-induced lung tropic cancer metastasis. *Int J Cancer* 144,3138–3145.

Mori S, Takahashi HK, Yamaoka K, Okamoto M, Nishibori M. (2003). High affinity binding of serum histidine-rich glycoprotein to nickel-nitrilotriacetic acid: The application to microquantification. *Life*

Sci 73,93–102.

Sakaguchi M, Watanabe M, Kinoshita R, Kaku H, Ueki H, Futami J, Murata H, Inoue Y, Li SA, Huang P., et al. (2014). Dramatic increase in expression of a transgene by insertion of promoters downstream of the cargo gene. *Mol Biotech* 56,621–630.
